# The Rare *Richardsitas* Betsch (Collembola, Symphypleona, Sminthuridae): A New Species from Australia with Comments on the Genus and on the Sminthurinae

**DOI:** 10.3390/insects11080519

**Published:** 2020-08-11

**Authors:** Gleyce da S. Medeiros, Penelope Greenslade, Bruno C. Bellini

**Affiliations:** 1 Departamento de Botânica e Zoologia, Laboratório de Collembola, Centro de Biociências, Universidade Federal do Rio Grande do Norte—UFRN. BR 101, Lagoa Nova, Campus Universitário, Natal 59072-970, Brazil; 2 School of Science, Psychology and Sports, Federation University, Ballarat, VIC 3353, Australia; p.greenslade@federation.edu.au; 3 Department of Biology, Australian National University, GPO Box, Canberra ACT 0200, Australia

**Keywords:** chaetotaxy, Oceania, Sminthuroidea, survey, *Temeritas*-group

## Abstract

*Richardsitas* Betsch is a small genus of Sminthurinae with only two species described so far, both from Madagascar. It resembles other Sminthurinae with long antennae, especially *Temeritas* Richards. Here we provide the first record of *Richardsitas* from Australia, *Richardsitas subferoleum* sp. nov., which is similar to *R. najtae* Betsch and *R. griveaudi* Betsch in males’ large abdomen chaetotaxy and presence of tenent-hairs on tibiotarsi II–III, but lacks mucronal chaeta and has 28 segments on the fourth antennal segment plus a unique pair of sensilla on the second. We also provide an updated genus diagnosis to *Richardsitas*, a key to its species, a discussion of the affinities of *Temeritas* and *Richardsitas* to other Sminthurinae, and an updated key to this subfamily.

## 1. Introduction

The family Sminthuridae Lubbock, 1862 [1] has about 260 nominal species in 32 genera and represents one of the most common and widespread groups of Symphypleona [2,3,4]. It comprises three subfamilies of which the Sminthurinae, Lubbock, 1862 is the largest, with about 180 species described in 12 extant and seven extinct genera [2,4,5,6,7,8,9]. The diagnosis of Sminthurinae was recently updated by Zeppelini et al. [9] and its main features are long antennae, mostly longer than the body; fourth antennal segment with well-marked subsegments; tibiotarsi with more than six chaetae on distal whorl; ungues without cavity; absence of neosminthuroid chaetae on the parafurcal area except in *Keratosminthurus* Zeppelini, 2020 in Zeppelini et al. 2020 [9]; dens ventrally usually with more than 9 chaetae [2,5,9]. The Sminthurinae fauna is poorly understood in Oceania and there are only four recorded species from Australia in two different genera: *Sminthurus viridis* (Linnæus, 1758) [10], *Temeritas denisii* (Womersley, 1934) [11], *T. regalis* (Womersley, 1939) [12]; *T. isabellae* Greenslade, 2013 [13,14].

*Richardsitas* Betsch, 1975 [15] is a small genus of Sminthurinae with only two described species, both from Madagascar: *R. griveaudi* Betsch, 1977 [16] and *R. najtae* Betsch, 1975 [15]. It is similar to *Temeritas* Richards, 1963 in Delamare Deboutteville and Massoud, 1963 [17], *Galeriella* Ćurčić and Lučić, 2007 in Ćurčić et al. 2007 [18] and *Keratosminthurus*, as well as species with long antennae of *Sminthurus* Latreille, 1802 [19], *Novokatianna* Salmon, 1944 [20], *Spatulosminthurus* Betsch and Betsch-Pinot, 1984 [21] and *Pararrhopalites* Bonet and Tellez, 1947 [22]. Such taxa have several subsegments (mostly 18 or more) on Ant IV, a metatrochanteral spine (except for *Sminthurus* and *Spatulosminthurus*), and mainly share a similar dental chaetotaxy [2,5,9,18,20,22,23,24]. However, *Richardsitas* is unique in its strong sexual dimorphism regarding the dorsal chaetotaxy of the large abdomen of the males, combined with capitate tenent-hairs on second and third pairs of legs [5,15,16]. Among the Sminthurinae, *Richardsitas* appears most similar to *Temeritas* as noted by Betsch [5,15], and females of both genera can be only clearly distinguished by presence/absence of capitate tenent-hairs. 

Herein we describe in detail the first species of *Richardsitas* from Australia and update the generic diagnosis. We also provide a key to *Richardsitas* species and discuss its affinities with other Sminthurinae genera. Finally, based on our survey of the Sminthurinae, we provide an updated key to the extant genera.

## 2. Materials and Methods

The specimens were preserved in ethanol, cleared in Nesbitt’s solution, and mounted in glass slides using Berlese’s medium. Morphological studies and raw drawings were made with a Leica DM750 microscope with an attached drawing tube. Photographs were taken with the same microscope, with a Leica MC170 HD camera using LAS V. 4.12 software. Final figures were improved and organized in plates using CorelDraw X8 software. Type series was deposited at the South Australian Museum, Adelaide, Australia, under the acronym of SAMA.

The terminology used in descriptions follows Betsch and Waller [25] for head and large abdomen chaetotaxy and Betsch [26] for small abdomen chaetotaxy, using as a model the species in Medeiros and Bellini [24]; Fjellberg [27] for labial palp papillae and Cipola et al. [28] for labral chaetotaxy. 

The abbreviations and symbols used are: Abd—abdominal segment; Ant—antennal segment; Th—thoracic segment. Chaetae present or absent are marked with white arrows, unpaired chaetae on frontal head and trunk are marked with a ‘*’ on drawings, extra chaetae on head without clear homologies are circled. Head, trunk (thorax + abdomen), and furcal chaetotaxy are given by half body in the text description. The chaetal labels are marked in bold in the text. 

## 3. Results

### 3.1. Taxonomic Summary and Genus Diagnosis

Order Symphyleona Börner, 1901 [29]
Superfamily Sminthuroidea Bretfeld, 1994 [30]
Family Sminthuridae Lubbock, 1862 [1]
Subfamily Sminthurinae Lubbock, 1862 [1]
Genus *Richardsitas* Betsch, 1975 [15]

*Diagnosis*. Specimens pigmented. Antennae longer than body length; Ant IV longer than Ant I–III combined, with 28–30 subsegments; Ant III apical organ sensory rods apart, in independent shallow invaginations. Eyes 8 + 8. Head frontal area with at least 3 pairs of spine-like chaetae; post antennal chaeta absent. Trochanter III with five normal chaetae plus one posterior spine. Tibiotarsi I–III with normal smooth or slightly serrated chaetae, tibial oval organs absent; capitate tenent-hairs present on tibiotarsi II–III; posterior and anterior pretarsal chaetae present. Ungues lanceolate without cavity or tunica, unguiculi with the apical filament. Large abdomen with anterior and posterior dorsal spines, with typical smooth or slightly serrated chaetae plus slender spines in females, and with 3–4 fields of peculiar short candle-shaped or spine-like chaetae in males. Bothriotrichia **A–D** present, **A–C** misaligned. Neosminthuroid chaetae absent on parafurcal area (furcal basis). Dens with 13 ventral chaetae, their formula as 1:1:2:2:2:2:3 from the basis to the apex. Mucro slender, with narrow apex, with both edges serrated. Mucronal chaeta present or absent (adapted from Betsch [5,15,16]).

Type species. *Richardsitas najtae* Betsch, 1975 [15].

### 3.2. Richardsitas subferoleum sp. Nov. 

**Figure 1:**
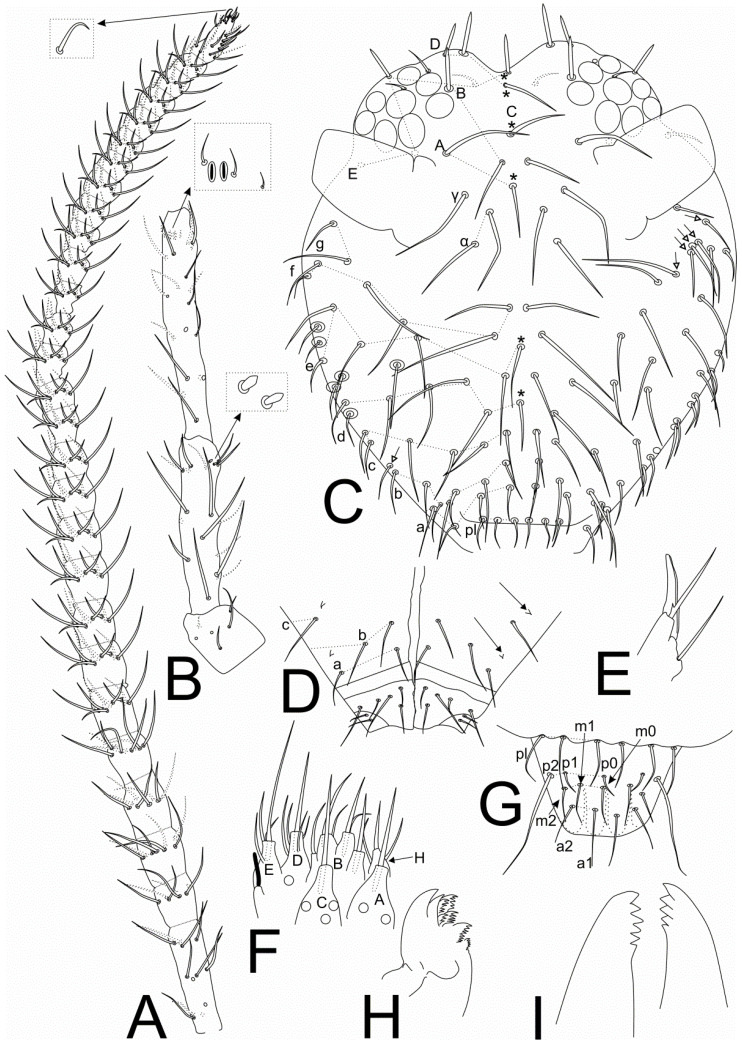
*Richardsitas subferoleum* sp. nov. head: (**A**) left Ant IV, arrow points to apical curved chaeta; (**B**) left Ant I–III, arrow on Ant II points to two modified apical sensilla, arrow on Ant III points to apical organ of Ant III; (**C**) frontal head chaetotaxy, circles = extra chaetae without clear homologies, * = unpaired chaetae, white arrows point to chaetae present or absent; (**D**) ventral head chaetotaxy, arrows point to cuticular spines; (**E**) right maxillary outer lobe and sublobal plate; (**F**) labial palp papillae (left side) and proximal chaetae (circles), lateral process in black; (**G**) prelabral and labral chaetae; (**H**) left maxilla capitulum; (**I**) right and left apices of mandibles (incisive teeth).

**Figure 2:**
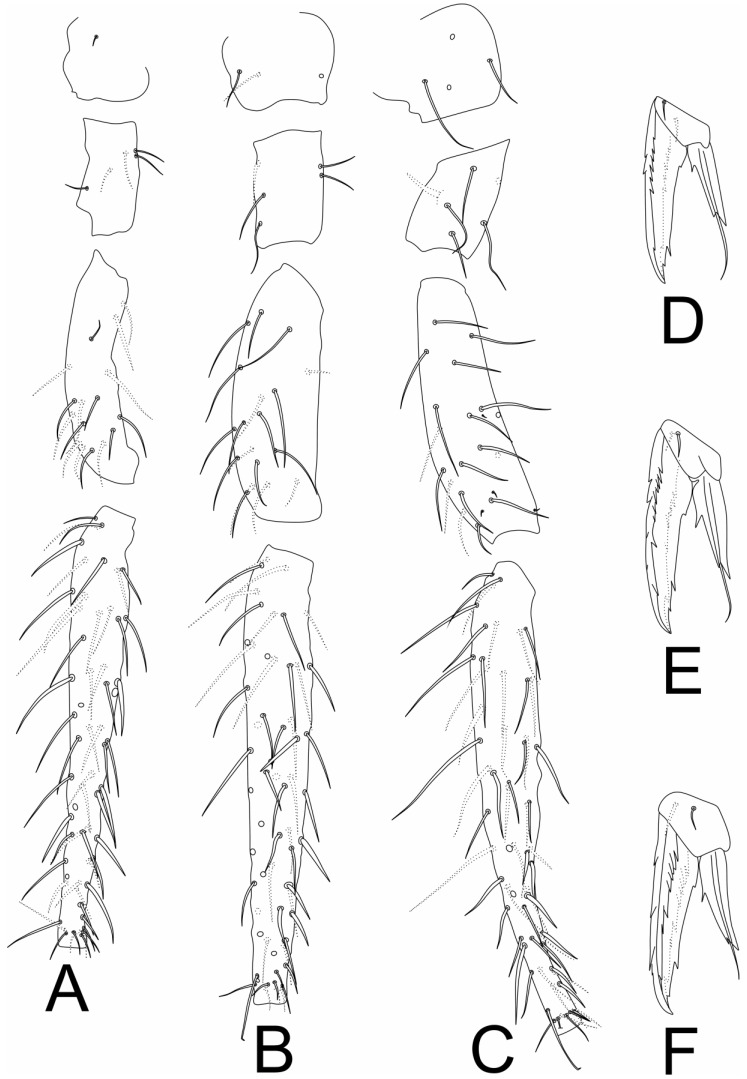
*Richardsitas subferoleum* sp. nov. legs: (**A**) leg I; (**B**) leg II; (**C**) leg III; (**D**) foot complex I; (**E**) foot complex II; (**F**) foot complex III.

**Figure 3:**
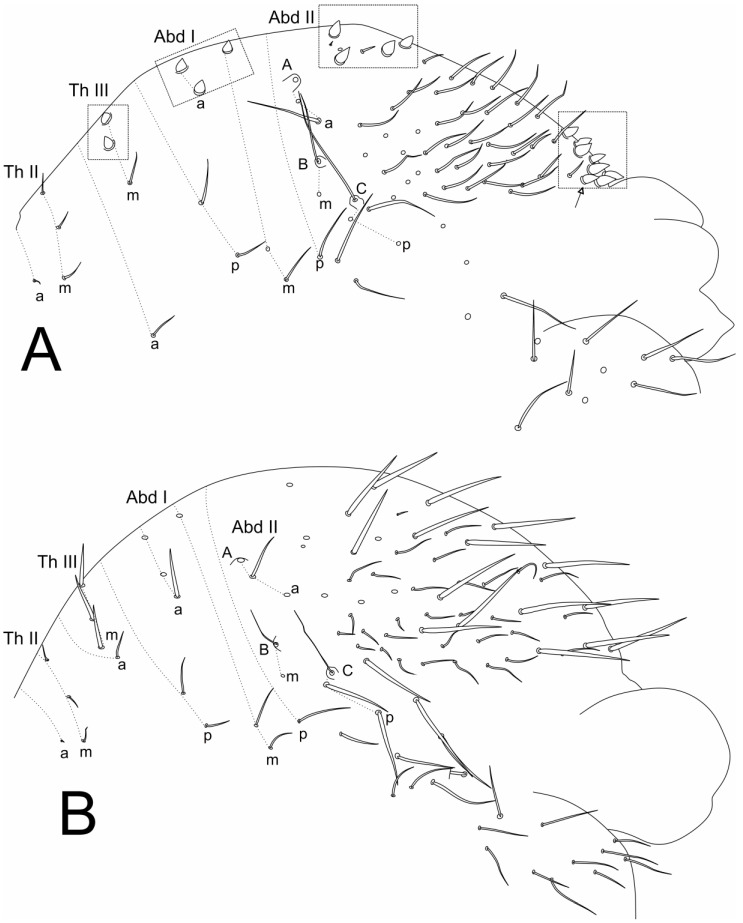
*Richardsitas subferoleum* sp. nov. large abdomen: (**A**) male, squares highlight fields of short candle-shaped chaetae on dorsum; (**B**) female.

**Figure 4:**
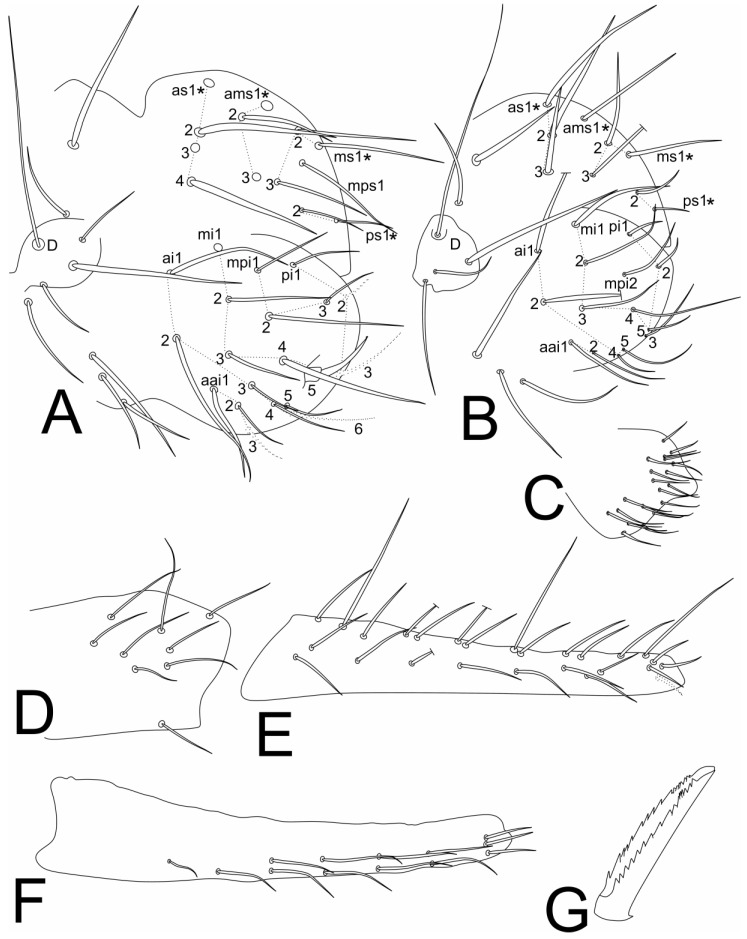
*Richardsitas subferoleum* sp. nov. small abdomen and furca: (**A**) chaetotaxy of small abdomen of female; (**B**) chaetotaxy of small abdomen of male (* = unpaired chaetae); (**C**) genital plate of male (lateral view); (**D**) manubrium (lateral view); (**E**) dorsal dens chaetotaxy; (**F**) ventral dens chaetotaxy; (**G**) mucro.

**Figure 5:**
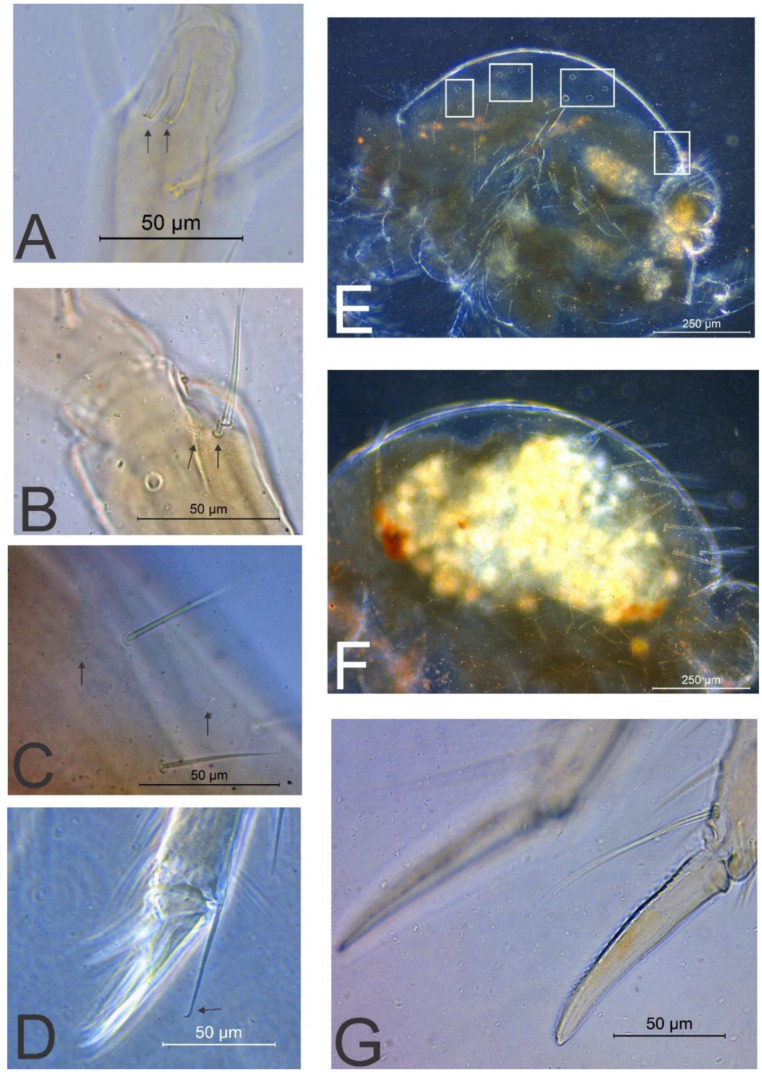
*Richardsitas subferoleum* sp. nov. photographs: (**A**) apex of Ant III, arrows point to apical organ sensory rods; (**B**) apex of Ant II, arrows point to small sensilla; (**C**) ventral head, arrows point to cuticular spines; (**D**) foot complex III, arrow points to capitate tenant-hair; (**E**) male large abdomen, squares highlight fields of short candle-shaped chaetae on dorsum; (**F**) female large abdomen; (**G**) mucro.

Figure 1, Figure 2, Figure 3, Figure 4 and Figure 5, Table 1.

*Type material.* Holotype: male on slide SAMA (voucher code VC36): Western Australia, Barrow Island, 20°47′52.8″ S, 115°24′21.6″ E, 15.iii.2006, pitfall-trap, S. Callan coll. Paratypes on slides SAMA (voucher code VC36): one female, same slide as the holotype; two females and one male (headless) on the same slide; one subadult male on a single slide, same data as holotype.

*Diagnosis.* Antennal segment IV with 28 subsegments, Ant II with two apical modified sensilla. Male with 4 zones with a total of 16–17 short candle-shaped dimorphic chaetae. Dens with 26 dorsal chaetae. Mucronal chaeta absent.

*Description.* Body (head + trunk) length of type series ranging between 0.8–1.37 mm, males average 0.87 mm, females average 1.27 mm, type series average 1.11 mm, holotype (male) with 0.95 mm. Habitus typical of the genus. Body colour in ethanol mottled pink, antennae mostly dark mauve, distal third of Ant III, and distal half of Ant IV whitish. Body chaetae slightly serrated and acuminate.

Head (Figure 1 and Figure 5A–C). Antennae longer than the body, with 1.52 mm in the holotype. Holotype antennal segments ratio Ant I:II:III:IV as 1:2.4:3.44:10.2. Ant IV with 28 subsegments in males and females, subsegment 1 with 12 chaetae, subsegment 2 with 7 chaetae, subsegments 3–4 with 8 chaetae each, subsegment 5 with 9 chaetae, subsegments 6–27 with 10 chaetae each, subsegment 28 with about 23 chaetae, one apical chaeta curved (Figure 1A). Ant III with 20 chaetae, apical organ typical with two sense rods inside two separate invaginations, surrounding subapical microsensillum present (Figure 1B and Figure 5A). Ant II with 17 chaetae, five longer and thicker, plus two small modified sensilla at the apex (Figure 1B and Figure 5B). Ant I with six chaetae (Figure 1B). Head length (eyes to mouth) of holotype 0.49 mm. Eyes 8 + 8 (Figure 1C). Clypeal area **a–g** lines with 6/9/7/5(+1)/5(+1)/6/2 chaetae respectively, six extra chaetae with unclear homologies (circled), the right side of the head of the holotype with five extra chaetae near **g** line field (Figure 1C). Interantennal area with only **α** and **γ** lines with 2 and 1 chaetae respectively; frontal area with **A–E** lines with 1(+1)/2/0(+1)/2(+1)/4(+1) chaetae respectively; 1, 2 and 1(+1) spiniform chaetae in lines **B**, **D** and **E** respectively; 2 interocular chaetae present (Figure 1C). Ventral head chaetotaxy as in Figure 1D, ventral groove surrounded by 2 chaetae on each side; lateral postlabial fields with 2 pairs of cuticular spines (Figure 1D and Figure 5C); labial basomedian field with 5 chaetae, basolateral field with 2 chaetae (Figure 1D). Maxillary outer lobe developed, with basal chaeta slightly smaller than the apical, both smooth, apical chaeta with internal proximal barb; sublobal plate entire, compressed laterally, lacking chaeta-like appendages (Figure 1E). Labial palp papillae as in Figure 1F with 6 proximal chaetae, formula of guard chaetae of each papilla as **H**(2), **A**(0), **B**(5), **C**(0), **D**(4), **E**(3) + blunt lateral process not reaching papilla **E** base. Labral chaetotaxy with 3 **pl**, 2(+1) **p**, 2(+1) **m** and 2 **a** chaetae, **p0–1** reduced, **p2** longer than others, labrum with 4 labial crests separated by 3 long grooves, reaching **m0–1** chaetae (Figure 1G). Maxilla typical, with six lamellae (Figure 1H). Mandibles asymmetrical with 5–6 incisive apical teeth (Figure 1I).

Legs (Figure 2). Coxa I with 1 chaeta; trochanter I with 5 chaetae; femur I with 16 chaetae, one on proximal half reduced; tibiotarsus I with 59 chaetae, distal whorl with 9 chaetae (Figure 2A). Coxa II with 3 chaetae; trochanter II with 5 chaetae; femur II with 18 chaetae, one on proximal half reduced; tibiotarsus II with 59 chaetae, distal whorl with 8 normal chaetae plus 1 capitate tenant-hair (Figure 2B). Coxa III with 4 chaetae; trochanter III with 5 regular chaetae plus 1 trochanteral spine; femur III with 18 regular chaetae plus 4 microchaetae; tibiotarsus III with 61 chaetae, distal whorl with 8 chaetae plus 1 capitate tenant-hair (Figure 2C and Figure 5D). Tibiotarsi I–III lacking oval organs (Figure 2A–C). Foot complexes I–III with two pretarsal chaetae (Figure 2D–F); ungues without tunica or cavity with 3 unpaired teeth, 1 proximal and 2 subapical, lateral and dorsal margins serrated. Unguiculi I–II main lamellae about ½ to ⅔ of the ungues length, with 2 internal and 1 apical teeth, filament reaching the unguis tip in unguiculus I and slightly smaller in unguiculus II, unguiculus III main lamellae about ⅔ the ungues length, with two internal and one apical teeth, filament not reaching the tip of unguis (Figure 2D–F).

Trunk (Figure 3, Figure 4A–C and Figure 5E–F). Trunk length of holotype (male) 0.84 mm. Large abdomen (Figure 3 and Figure 5E–F): thorax continuous with abdomen, without any visible segmentation or constrictions (Figure 3). Male: Th II with 1 **a** and 3 **m** chaetae; Th III with 1 **a**, 3 **m** and 2 **p** chaetae; Abd I with 2 **a**, 3 **m** and 1 **p** chaetae; bothriotrichia **A, B** and **C** present in Abd II and misaligned; bothriotrichia **A** with 2 (**a**), **B** with 1 (**m**) and **C** with 2 (**p**) accessory chaetae each, respectively; large abdomen with 4 zones (on Th III, Abd I, III–IV) with short candle-shaped chaetae with 2, 3, 4 and 7–8 chaetae, respectively; parafurcal area with 10 main normal chaetae (Figure 3A and Figure 5E). Female: Th II with 1 **a** and 3 **m** chaetae; Th III with 1 **a**, 3 **m** and 2 **p** chaetae; Abd I with 3 **a**, 3 **m**, and 1 **p** chaetae; bothriotrichia **A, B** and **C** present in Abd II and misaligned; bothriotrichia **A** with 2 (**a**), **B** with **1** (**m**) and **C** with 2 (**p**) accessory chaetae each, respectively; large abdomen with about 16 long spine-like chaetae, short candle-shaped chaetae seen in males completely absent; parafurcal area with 11 main normal chaetae (Figure 3B and Figure 5F). Small abdomen of female in Figure 4A, with bothriotrichium **D**; dorsal anal valve with **as1–4**, **ams1–3**, **ms1–3**, **mps1** and **ps1**–**2** chaetae, **as1**, **ams1**, **ms1** and **ps1** unpaired; ventral anal valves each with **aai1–3**, **ai1–6**, **mi1–5**, **mpi1–3** and **pi1–3** chaetae; **mi5** as subanal appendage curved toward the anus opening, smooth, thick and apically with serrated tip. Small abdomen of male in Figure 4B, with bothriotrichium **D**; dorsal anal valve with **as1–3**, **ams1**, **ms1–3** and **ps1–2** chaetae, **as1**, **ams1**, **ms1** and **ps1** unpaired; ventral anal valves each with **aai1–2**, **ai1–2**, **4–5**, **mi1–5**, **mpi2** and **pi1–3** chaetae. Genital plate of male with about 20 chaetae on each side (Figure 4C); genital plate of female not seen.

Abdominal appendages (Figure 4D–G and Figure 5G). Ventral tube corpus apparently lacking any chaeta, with a pair of warty sacs. Tenaculum with 3 teeth on each ramus and 1 + 1 apical chaetae on corpus. Furcal size length in holotype as: manubrium = 0.31 mm; dens = 0.34 mm; and mucro = 0.11 mm. Manubrium with 8 dorsal and one ventral chaetae (Figure 4D); dens dorsally (posteriorly) with 26 chaetae, one proximal, one median and one distal longer than others (Figure 4E); dens ventrally (anteriorly) with 13 chaetae, with the following formula from proximal to distal region: 1:1:2:2:2:2:3 (Figure 4F); mucro with narrow apex, with both edges serrated, with about 17 teeth on each edge, mucronal chaeta absent (Figure 4G and Figure 5G). Ratio mucro: dens: manubrium in holotype 1:3.18:2.82.

*Etymology.* From Latin, *subfero* = to endure, to tolerate; and *oleum* = oil. The new species has survived widespread oil extraction on Barrow Island for many years.

*Distribution, Habitat and Conservation.* Barrow Island is Class A Sanctuary protected by Western Australian legislation. It is a continental island being only 56 km offshore the Western Australian coast and with about 236 km² of total area. Most of the island is covered with hummock grassland (*Triodia* sp.) with scattered shrubs, herbs and rare *Ficus* trees [31]. It is in the wet/dry tropics with rain falling almost entirely in the summer months and then generally in short, sharp heavy downpours. The climate of the area is “BWh” following the Köppen-Geiger climate classification, which means an arid main climate with desert-like precipitation and overall “hot arid” temperatures [32]. 

Although Barrow Island is a sanctuary, it is inserted in a prolific oil field area. Its biota has been exposed to commercial oil extraction for the past decades, and more recently to natural gas processing as well [31].

*Richardsitas subferoleum* sp. nov. type specimens were all collected in pitfall traps after a rare heavy rainfall of nearly 50 mm within 24 hours in March 2006 and on none of the other seven sampling occasions from 2005 to 2012. The only other known local springtail species that responded in a similar way to a significant rainfall event was *Pygicornides* sp. The collection sites in 2006 were all clustered around the location of the planned gas plant before construction. How much of the original vegetation of hummock grassland with scattered shrubs and herbs remains undisturbed now after construction, is not known. A sole collection was made in May 2007 and a few more specimens of *Richardsitas subferoleum* sp. nov. were found at that time, near to a new airfield. Again, the vegetation was largely hummock grassland with some native grasses and shrubs. It is likely that *Richardsitas subferoleum* sp. nov. is widespread on the island but is only active after a significant rainfall event mainly in summer. 

*Remarks. Richardsitas subferoleum* sp. nov. resembles *R. najtae* and *R. griveaudi* by its *Temeritas*-like habitus, presence of dimorphic short candle-shaped chaetae on dorsal large abdomen of males, presence of capitate tenant-hairs on tibiotarsi II–III, absence of oval organs on all tibiotarsi and ventral chaetotaxy of dens following the formula 1:1:2:2:2:2:3. However the new species is unique in its combination of 28 antennal subsegments on Ant IV (30 in the other two species), 4 zones of short candle-shaped chaetae on large abdomen of males (3 in *R. griveaudi*) and mucronal chaeta absent (present in the other two species). The new species also differs in the reduced number of dorsal short candle-shaped chaetae of males (16–17 on each side), while *R. griveaudi* has about 26 and *R. najtae* about 58. The overall morphology of such chaetae is constant in the new species while in both *R. griveaudi* and *R. najtae* there are smaller and larger chaetae combined at least in the posterior zone of the large abdomen. Lastly, the new species has a peculiar organ on dorsal Ant II with two modified sensilla, absent in the other two species of *Richardsitas.* The main differences between the three species are summarized in Table 1.

*Richardsitas najtae* and *R. griveaudi* were recorded from semi-arid forests in south and southwest Madagascar, at the same latitude as Barrow Island [5,15,16]. This may be significant especially as both islands experience to some extent the same climate, especially in the summer rainfall. 

### 3.3. Identification Key and Distribution of Richardsitas Species

1. Mucronal chaeta absent; Ant IV with 28 subsegments; Ant II with 2 dorso-apical short sensilla; males with 16–17 short candle-shaped chaetae distributed in 4 zones of dorsal large abdomen … *Richardistas subferoleum* sp. nov.; Australia
  ‒Mucronal chaeta present; Ant IV with 30 subsegments; Ant II lacking modified short sensilla; males with about 26 or more short candle-shaped or spine-like chaetae distributed in 3–4 zones of the dorsal large abdomen … 2
2. Males with about 26 short candle-shaped chaetae distributed in 3 zones of the dorsal large abdomen … *R. griveaudi* Betsch, 1977 [16]; Madagascar
  ‒Males with about 58 short candle-shaped or spine-like chaetae distributed in 4 zones of dorsal large abdomen … *R. najtae* Betsch, 1975 [15]; Madagascar

## 4. Discussion

### 4.1. Remarks on the Distribution and Morphology of the Richardsitas Species

**Figure 6:**
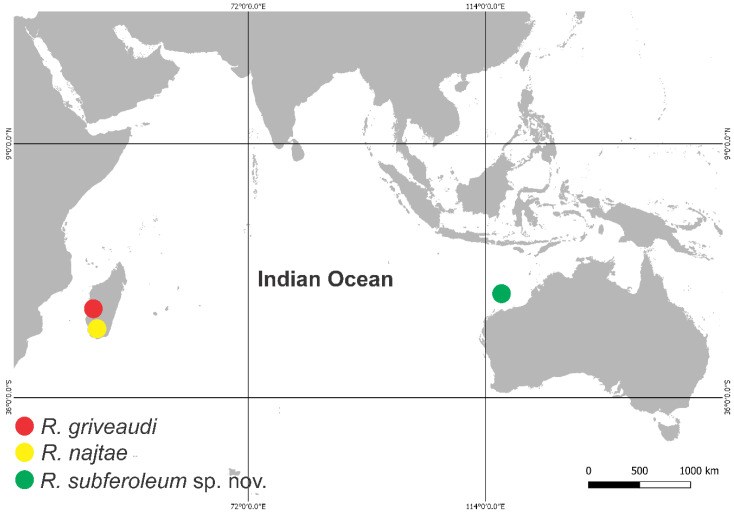
Distribution of *Richardsitas* species: *R. griveaudi* (red) and *R. najtae* (yellow) are known from Madagascar; *R. subferoleum* sp. nov. is only known from Barrow Island, Western Australia.

The unusual morphology of the three *Richardsitas* species, with *Temeritas*-like habitus combined with a single capitate tenent-hair on tibiotarsi II and III and males with short candle-shaped or spine-like chaetae on dorsal large abdomen, is unmatched among the subfamily Sminthurinae (Table 2). It strongly suggests the genus is monophyletic, although a rigorous phylogenetic analysis must confirm this hypothesis. In this sense the disjunct distribution of *Richardsitas* in Madagascar and Western Australia may point at least to two different scenarios: a Gondwanan origin to the genus, about at least 100 million years ago with the break of East Gondwana; or a more recent colonisation through the Indian Ocean, similarly to the model proposed by Christiansen and Bellinger [33] to Hawaii colonisation. If the first hypothesis is true, it is highly possible that relict populations of *Richardsitas* were isolated in Madagascar, Australia (as in Barrow Island), and other localities after the breakup of the former Gondwana supercontinent. In either case, considering its distribution, it is possible *Richardsitas* has a wider distribution around the Indian Ocean (Figure 6).

Except for the previously discussed features, the overall morphology of *Richardsitas* is remarkably similar to *Temeritas*, as originally stated by Betsch [15], and antennal morphology supports that the first genus is similar to *Temeritas stricto sensu* ingroup [24]. Other features shared by *Richardsitas* and *Temeritas* are the presence of 8 + 8 eyes, absence of postantennal chaeta, presence of trochanteral spine, smooth **D** bothriotrichium and dens with 13 ventral (anterior) chaetae (Table 2). These characteristics, including the long subsegmented antennae, are also found in *Galeriella* and *Keratosminthurus*, with exception of eyes being absent in *Galeriella* (dental ventral chaetotaxy unknown in this genus) and head dimorphic features in *Keratosminthurus* [9,18]. Because of these similarities, we consider such Sminthurinae genera are possibly closely related within *Temeritas*-group. Specialisations like loss of eyes and body pigments in *Galeriella* are related to a troglobiont way of life. We are not including in this group other genera like *Janusius* Bretfeld, 2010 [34], *Sminthurus* and *Spatulosminthurus*, as they do not share the metatrochanteral spine, a feature which may be significant to separate them from other Sminthurinae [2] (Table 2). Because of variable morphology we did not include *Pararrhopalites* in *Temeritas*-group; most of its species have fewer than 15 subsegments on Ant IV [23,24]. Nevertheless, at least a few species of *Pararrhopalites* resemble *Temeritas ormondae*-group [24].

Our diagnosis of *Richardsitas* mainly fits the one proposed by Betsch [5,15], with some additions, especially the variation of Ant IV subsegments and presence or absence of mucronal chaeta. Such differences are considered as interespecific variations within other Sminthurinae genera, such as in *Temeritas* (Table 2). On the other hand, the lack of further data concerning labrum, labium, ventral head, legs, among other features of *R. najtae* and *R. griveaudi*, prevents us providing additional notes on differences/diagnostic attributes of *Richardsitas*. For instance, none of Betsch’s descriptions show modified sensilla on the Ant II as seen in *Richardsitas subferoleum* sp. nov. Betsch [15,16] described Ant II and made notes on antennae of *R. najtae* and *R. griveaudi*, and so we consider both lack such an organ. 

### 4.2. Remarks on Some Sminthurinae Genera

According to Bernard and Wynne [35] the diagnoses of subfamilies of Sminthuridae are partially supported by overlapping taxonomic characters, such as the presence, absence or shape of the neosminthuroid chaetae in the parafurcal area, ventral dens chaetotaxy and number of Ant IV subsegments. The unreliability of subfamily diagnoses makes the placing of some genera like *Keratosminthurus* uncertain [9]. The same applies to other Sminthurinae genera, which in some cases cannot clearly be distinguished from each other (Table 2). A large study concerning the evolution and validity of internal taxa of Sminthurinae is needed to better delimit which morphological features are of phylogenetic significance in this group. 

Regarding the extinct taxa listed as Sminthurinae in Bellinger et al. [4], at least one genus may not belong to the subfamily, *Brevimucronus* Christiansen and Pike, 2002 [6]. The description of its antennae morphology and measurement are ambiguous, and the genus apparently is related to Dicyrtomidae. The authors described the fourth antennal segment possibly bearing a large apical bulb, but it is most likely a reduced Ant IV since it has a few chaetae. If this is true, then the antennae are elbowed between Ant II and III, as seen in extant Dicyrtomidae (see Christiansen and Pike [6] (p. 180, Figure 36)) [6]. Other extinct genera are similar to Sminthurinae, but there are several uncertainties about their morphology (see Table 2). At least the identity of extinct Sminthurinae genera without the metatrochanteral spine should be taken with caution as an incomplete understanding of morphology could hide taxa more related to other subfamilies of Sminthuridae or even other families of Symphypleona. 

Based on our survey of the Sminthurinae we provide the following key to its extant genera.

### 4.3. Identification Key and Distribution of Extant Sminthurinae Genera

1. Metatrochanteral spine absent ... 2
  –Metatrochanteral spine present ... 4
2. Capitate tenent-hairs absent on tibiotarsi ... *Sminthurus* Latreille, 1802 [19]; Holarctic*
  –Capitate tenent-hairs present on tibiotarsi ... 3
3. Female’s subanal appendage short, chaeta-like, oval or leaf-like; male’s genital plate with normal granules ... *Spatulosminthurus* Betsch and Betsch-Pinot, 1984 [21]; Palaearctic
  –Female’s subanal appendage long and chaeta-like; male’s genital plate with short cuticular points … *Janusius* Bretfeld, 2010 [34]; Holarctic
4. **D** bothriotrichium ciliate ... *Austrosminthurus* Delamare Deboutteville and Massoud, 1963** [17]; Argentina
  –**D** bothriotrichium smooth ... 5
5. A single neosminthuroid chaeta present on parafurcal area; males with two horn-like chaetae on apical Ant III ... *Keratosminthurus* Zeppelini, 2020 [9]; Brazil
  –Neosminthuroid chaeta on parafurcal area absent; males lacking horn-like chaetae on apical Ant III ... 6
6. Male’s dorsal large abdomen with 3–4 zones of short candle-shaped or spine-like chaetae; one capitate tenent-hair present on tibiotarsi II–III ... *Richardsitas* Betsch, 1975 [15]; Madagascar, Australia
  –Male’s dorsal large abdomen without zones of modified chaetae; tibiotarsi without tenent-hairs ... 7
7. Postantennal chaeta present; dorsal head and large abdomen with long rough often blunt chaetae; large abdomen posteriorly with one pair of cuticular glands ... *Allacma* Börner, 1906 [36]; Holarctic
  –Postantennal chaeta absent; dorsal head and large abdomen chaetotaxy with regular and/or spine-like chaetae; large abdomen posteriorly without cuticular glands ... 8
8. Large abdomen dorso-posterior spines present ... 9
  –Large abdomen dorso-posterior spines absent ... 10
9. Ant IV with 15–18 subsegments; head and bothriotrichia areas of cuticle with complex girandole-like granules ... *Caprainea* Dallai, 1970 [37]; Palaearctic
  –Ant IV mostly with 9–14 subsegments, rarely with 15; head and body cuticle without remarkable different granules … *Pararrhopalites* Bonet and Tellez, 1947 [22]; Holotropical
10. Ant IV with 13–15 subsegments; males with a well-developed clasping organ on ventral Abd VI ... *Novokatianna* Salmon, 1944 [20]; New Zealand
  –Ant IV with 18 or more subsegments; males devoid of a clasping organ on ventral Abd VI ... 11
11. Eyes and body pigment absent; cave species ... *Galeriella* Ćurčić and Lučić, 2007 [18]; Bosnia-Herzegovina
  –8+8 eyes present, specimens pigmented; surface species ... *Temeritas* Richards, 1963 [17]; Holotropical

* Species in the Southern Hemisphere are introduced.

** *Genus inquirenda*. Its sole species was described based in a single specimen lacking the Ant IV. Its reduced chaetotaxy strongly suggests the studied specimen (male) is a juvenile, as pointed by Bretfeld [2].

## 5. Conclusions

With the description of *R. subferoleum* sp. nov., there are now three described species of *Richardsitas*. Although the new species fits the genus diagnosis, it shows a remarkably reduced number of short candle-shaped chaetae on male’s dorsum, and a peculiar organ on apical Ant. II. The new species expands the genus distribution to Australia. *Richardsitas* morphology supports it is closely related to *Temeritas*, a Holotropical genus with species recorded from Madagascar and Australia as well. Sminthuridae subfamilies and genera’s diagnoses are partially based on overlapping features, especially among the Sminthurinae, and should be investigated under a wide phylogenetic analysis to better circumscribe them and to delimit which morphological features have phylogenetic significance.

## Figures and Tables

**Table 1:** Main characters of *Richardsitas* species.

Species	Colour	Ant IV Subsegments	Ant II ap. Sens	Dorsal Abdominal Zones of Modified Chaetae *	Heterogeneity of Abdominal Spines *	Number of Abdominal Spines *	Dens Dorsal (Posterior) Chaetae	Mucronal Chaeta
*R. griveaudi* [5,16]	pink to red	30	-	3	+	26	?	+
*R. najtae* [5,15]	red	30	-	4	+	58	26	+
*R. subferoleum* sp. nov.	pink	28	+	4	-	16–17	26	-

Legends: ap. Sens = modified apical sensilla; * = on males; + = present; - = absent; ? = unknown.

**Table 2:** Main diagnostic characters of Sminthurinae genera *sensu* Bellinger et al. 1996–2020.

Genera	Ant IV Subsegments	Eyes	Head Sexual Dimorphism	Post Antennal Chaeta	Abdominal Dorso-Anterior Spines	Abdominal Dorso-Posterior Spines	Dorsal Large Abdomen Sexual dimorphism	D Bothriotrichium	Neosminthuroid Chaetae on Large abdomen	Metatrochanteral Spine	Capitate Tenent-Hairs	Ungual Tunica	Dens Ventral Chaetae	Mucronal Chaeta
*Galeriella* ∆ [18]	32	-	-	-	-	-	-	Smooth	-	+	-	-	?	-
*Keratosminthurus* ∆ [9]	18–20	+	+	-	-	-	-	Smooth	+	+	-	+/-	12–13	-
*Richardsitas* ∆ [5,15,16]	28–30	+	-	-	+	+	+	Smooth	-	+	+	-	13	+/-
*Temeritas* ∆ [5,17,24]	18–46	+	-	-	+/-	-	-*	Smooth	-	+	-	+/-	13	+/-
*Allacma* [2,36]	12–15	+	-	+	-	-	-	Smooth	-	+	-	+/-	11–15	+
*Austrosminthurus* [5,17] ***	?	+	-?	-	?	-	-?	Ciliate	-?	+	-	+	13	+
*Caprainea* [2,37]	15–18	+	-	-	+	+	-	Smooth	-	+	-	+	About 13	+/-
*Janusius* [34,38]	18–26	+	+/-	+/-	-	-	-	Smooth	-	-	+	-	9–15	+/-
*Novokatianna* [5,20]	13–15	+	-	-	-	-	-	Smooth	-	+	-	?	?	+
*Pararrhopalites* [2,22,23] **	9–15	+/-	-	-	+/-	+	-	Smooth	-	+	-	+/-	7–14	+/-
*Sminthurus* [2,5]	14–30	+	+/-	+/-	-	-	-	Smooth	-	-	-	+/-	About 15	+/-
*Spatulosminthurus* [2,21]	14–25	+	+/-	+/-	-	-	-	Smooth	-	-	+	+/-	13–15	+/-
*Archeallacma* † [8]	14–15	+	?	?	-	-	?	?	?	?	-	+	?	-
*Brevimucronus* † [6]	-	+	?	?	?	?	?	?	?	-?	+	-	?	-?
*Grinnellia* † [7]	10	+	-?	-?	+	+	?	Smooth	-?	+	-	+/-	?	-?
*Katiannasminthurus* † [8]	14	+	?	?	+	+	?	?	-?	?	-	+	?	-
*Mucrovirga* † [7]	9–10	+	?	?	+?	+?	?	?	?	?	+	-	?	-?
*Sminthurconus* † [7]	12	+	?	?	+	+	?	?	?	+	-?	-	?	-?
*Sminthuricinus* † [7]	11–12	+	?	?	+	+	?	Smooth?	?	+	+	-	?	-?

Legends: [] = species references; ∆ = *Temeritas*-group; † = extinct; + = present; - = absent; ? = unknown/unclear; * = some species of *Temeritas* have sexual dimorphism on parafurcal area, the males can have plumose chaetae nearside the genital opening (see Medeiros and Bellini 2019); ** = here we did not consider *Parrarrhopalites indianus* Baijal and Argarwal, 1972 [39], since its antennae description is unclear; *** = *genus inquirenda*, see footnote of Sminthurinae genera key.
